# Selective versus non-selective NSAIDs as prophylaxis for heterotopic ossification following hip arthroplasty: a meta-analysis

**DOI:** 10.1186/s10195-022-00646-7

**Published:** 2022-07-09

**Authors:** Filippo Migliorini, Andrea Pintore, Alice Baroncini, Torsten Pastor, Frank Hildebrand, Nicola Maffulli

**Affiliations:** 1 Department of Orthopaedic, Trauma, and Reconstructive Surgery, RWTH University Hospital, Pauwelsstraße 30, 52074 Aachen, Germany; 2 Department of Medicine, Surgery and Dentistry, University of Salerno, 84081 Baronissi, SA Italy; 3 Department of Orthopaedic and Trauma Surgery, Cantonal Hospital of Lucerne, 6000 Lucerne, Switzerland; 4 School of Pharmacy and Bioengineering, Faculty of Medicine, Keele University, ST4 7QB Stoke on Trent, England; 5 Barts and the London School of Medicine and Dentistry, Centre for Sports and Exercise Medicine, Mile End Hospital, Queen Mary University of London, E1 4DG London, England

**Keywords:** Hip, Arthroplasty, Heterotopic ossification, NSAIDs, Selective, Non-selective

## Abstract

**Background:**

Some patients have demonstrated evidence of heterotopic ossification (HO) following total hip arthroplasty (THA). Selective and non-selective non-steroidal anti-inflammatory drugs (NSAIDs) are used as prophylaxis for HO following THA. This meta-analysis compared selective versus non-selective NSAIDs as prophylaxis for HO following THA.

**Material and methods:**

The present study was conducted according to the PRISMA 2020 guidelines. All the clinical investigations comparing selective versus non-selective NSAIDs as prophylaxis for HO following THA were accessed in February 2022. An assessment of the methodological quality and statistical analyses were performed through the risk of bias summary tool of the Review Manager 5.3 software (Cochrane Collaboration, Copenhagen). The modified Brooker staging system was used to rate the efficacies of the interventions.

**Results:**

Data from 8 studies and 1526 patients were collected. 60.8% were female. No difference was found in the sample size, mean age, and percentage of females between the two groups at baseline. No statistically significant difference was found between selective and non-selective NSAIDs in term of efficacy. 72% (1078 of 1502) of the patients were classified as Brooker 0, 21% (322 of 1502) as Brooker I, 5% (80 of 1502) as Brooker II, 1% (16 of 1502) as Brooker III, and 0.1% (2 of 1502) as Brooker IV.

**Conclusion:**

Selective and non-selective NSAIDs were equally effective when used as prophylaxis for HO following THA.

**Level of evidence:**

Level III, systematic review and meta-analysis.

## Introduction

In the absence of prophylaxis, the frequency of heterotopic ossification (HO) following total hip arthroplasty (THA) varies from 15 to 90% [1–5]. The exact cause and mechanism of bone formation after hip replacement remains unclear. Several approaches to reducing the occurrence of HO have been proposed, such as radiotherapy, non-steroidal anti-inflammatory drugs (NSAIDs), and diphosphonates [2, 5–8]. Evidence suggests that inhibition of the inflammation pathway may represent the underlying mechanism for ossification prevention [2]. The prophylactic effect of NSAIDs on HO was first documented when indomethacin was used as an analgesic after THA [9]. NSAIDs are typically divided into groups based on their cyclooxygenase (COX) selectivity: non-selective NSAIDs are directed to both COX-1 and COX-2, and selective NSAIDs are directed specifically to COX-2 [10]. Indomethacin is the NSAID most frequently used as prophylaxis for HO [11–13]. Other non-selective NSAIDs such as ketorolac, acetylsalicylic acid, meloxicam, naproxen, ibuprofen, and diclofenac have also been employed successfully [14–17]. Gastrointestinal complications are the most common reason for therapy discontinuation in patients treated with non-selective NSAIDs [18, 19]. Given their lack of interactions with platelet aggregation and gastrointestinal complications, selective NSAIDs are effective treatment alternatives to non-selective NSAIDs [4, 5, 20–24]. In selected patients, celecoxib is a valid alternative to non-selective NSAIDs, demonstrating efficacy, tolerability, and a lower rate of therapy discontinuation [20, 21, 25, 26]. Even rofecoxib was effective when used as prophylaxis for HO [7, 27]. However, many studies have shown an elevated risk of cardiovascular and renal complications with selective NSAID administration [4, 5, 20–22, 28]. The elevated cardiovascular risk was evidenced in patients treated with selective NSAIDs for longer than 6 months [22, 25]. However, whether the administration of selective NSAIDs for less than 20 days leads to an elevated risk of cardiovascular complication remains unclear [29]. Whether selective NSAIDs are equally as effective as non-selective NSAIDs for the prevention of HO following THA has also not been fully clarified. Therefore, a meta-analysis was conducted. This study compared selective versus non-selective NSAIDs as prophylaxis for HO following THA.

## Methods

### Eligibility criteria

All the clinical trials comparing selective versus non-selective NSAIDs as prophylaxis for HO following THA were accessed. Only studies with accessible full texts that are published in peer-reviewed journals were considered. Given the authors’ language capabilities, articles in English, German, Italian, French, and Spanish were eligible. Only prospective evidence level I and II studies, according to the Oxford Centre of Evidence-Based Medicine [30], were considered. Reviews, opinions, letters, and editorials were not considered. Animal, in vitro, biomechanical, computational, and cadaveric studies were all not eligible. Studies evaluating HO in locations other than the hip were not considered, nor were those evaluating procedures other than THA. Studies which evaluated radiation, hormonal therapy, or other experimental therapies were not considered. Only studies that evaluated the rate of HO following THA using the Brooker classification [31] in a clinical setting were eligible. Missing quantitative data for the outcome of interest warranted the exclusion of the study from the present investigation.

### Search strategy

This systematic review was conducted according to the 2020 Preferred Reporting Items for Systematic Reviews and Meta-Analyses (PRISMA) statement [32]. The PICO algorithm was preliminarily pointed out:

- P (population): patients following THA
- I (intervention): prophylaxis of HO
- C (comparison): selective versus non-selective NSAIDs
- O (outcomes): Brooker classification [31].

In February 2022, the following databases were accessed: Pubmed, Web of Science, Google Scholar, and Embase. No time constraints were used in the search. The following keywords were used in combination with the Boolean operators AND/OR: hip, replacement, arthroplasty, prosthesis, heterotopic, ossification, impingement, indomethacin, naproxen, NSAIDs, selective, non-selective, prostaglandin, cyclooxygenase, acetylsalicylic acid, celecoxib, meloxicam, COX-inhibitors, rofecoxib, ibuprofen, diclofenac.

### Selection and data collection

Two authors (A.P. and F.M.) independently performed the database search. All the resulting titles were screened and, if suitable, the abstract was accessed. The full texts of the abstracts that matched the topic were accessed. A cross-reference of the bibliographies of the full-text was also performed. Disagreements were debated, and the final decision was made by a third author (N.M.).

### Data extraction

Two authors (A.P. and F.M.) performed data extraction independently. Study generalities and the patient demographics were extracted: author, year, length of the follow-up, type and protocol of the treatment, number of patients, mean age, and percentage of women. The outcome of interest was to compare the rate of HO following THA between selective and non-selective NSAIDs. The modified Brooker staging system was used to rate the efficacy of the interventions. This classification differs from the original by an additional grade of 0, in which there is no sign of HO [33] (Table 1).

**Table 1:** Modified Brooker staging system

Class	Radiographic findings
Grade 0	No sign of heterotopic ossification
Grade I	Bony islands in the soft tissue around the hip
Grade II	Exophytes in the pelvis or proximal end of the femur with at least 1 cm between opposing bone surfaces
Grade III	Exophytes in the pelvis or proximal end of the femur with less than 1 cm between opposing bone surfaces
Grade IV	Bony ankylosis between proximal femur and pelvis

### Risk of study bias assessment

The risk of study bias was assessed by one author (A.P.) using Review Manager version 5.3 (Nordic Cochrane Collaboration, Copenhagen). The risk of bias graph was used to assess the methodological quality of the included studies. Allocation, randomization, blinding of the assessors, selective reporting, incomplete data, and an unknown source of bias were used for assessment. To assess the overall risk of publication bias, a funnel plot of the most reported endpoint was created and evaluated.

### Synthesis methods

The statistical analyses were conducted by the main author (F.M.) using the Review Manager software (RevMan 5.3, Nordic Cochrane Collaboration, Copenhagen). For dichotomic data, the Mantel–Haenszel method with an odds ratio (OR) effect measure was adopted. The confidence interval was set at 95% in all comparisons. Higgin’s *I*^2^ test was conducted to evaluate heterogeneity. Values of *I*^2^ of greater than 50% indicated that the analysis may be affected by substantial heterogeneity. The $\chi$^2^ test was conducted to evaluate whether data were statistically significant. *P* < 0.05 indicated statistically significant heterogeneity. A fixed model effect was adopted as default. If *I*^2^ > 50% and *P* $\chi$^2^ < 0.05, a random effect model was adopted. Egger’s linear regression was performed using the STATA MP software (version 16; StataCorp, College Station, USA) to assess the asymmetry of the funnel plot, with values of *P* < 0.05 indicating statistically significant asymmetry.

## Results

### Study selection

**Fig. 1:**
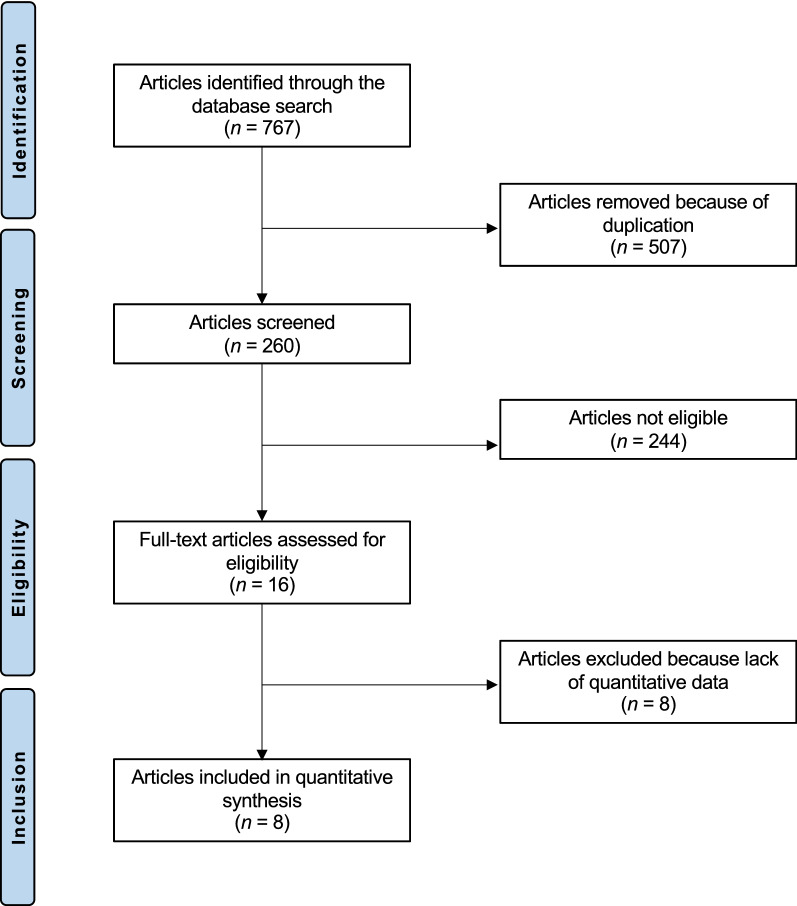
Flow chart of the literature search

The literature search resulted in 6023 articles. Of those, 767 focused on prophylaxis for HO following THA. Of those, 507 were excluded as they were duplicates. A further 252 articles were excluded as they did not match the eligibility criteria: the full text was not accessible or published in peer-reviewed journals (*N* = 11); language limitation (*N* = 8); poor level of evidence (*N* = 29); inappropriate study design (*N* = 47); locations other than the hip or procedures other than THA were evaluated (*N* = 94); the comparison was not between selective and non-selective NSAIDs (*N* = 51); the Brooker classification was not used (*N* = 4); quantitative data on the outcome of interest were missing (*N* = 8). Finally, eight studies were included in the present investigation. The literature search results are shown in Fig. 1.

### Risk of study bias assessment

**Fig. 2:**
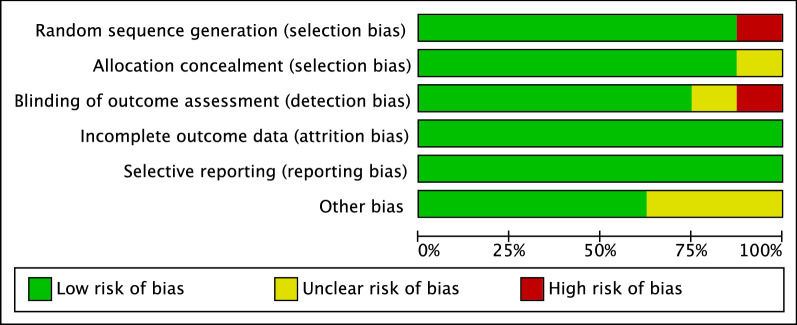
Methodological quality assessment

In the above-mentioned assessment of risk of bias, a very low risk of selection bias was evidenced. Similarly, the risk of attrition and reporting bias can be considered to be very low. The risk of detection bias scored low. This reflected the fact that randomization was present in most of the included studies. The risk of unknown bias was also moderate to low. Therefore, the methodological assessment of the bias in this work gave very good results. The Cochrane risk of bias summary tool is shown in Fig. 2.

### Risk of publication bias

**Fig. 3:**
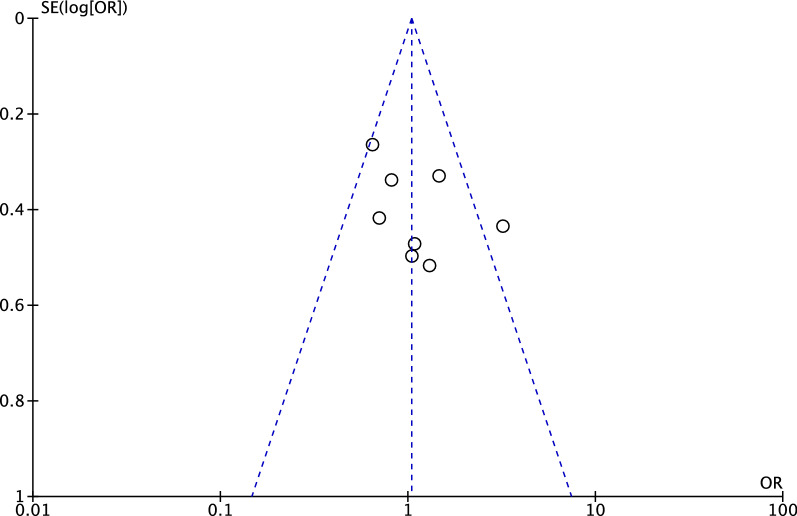
Funnel plot of the most reported outcome

A funnel plot of the most reported comparison (Brooker class 0) was created and evaluated (Fig. 3). The plot evidenced adequate symmetry. Egger’s test evidenced any statistically significant asymmetry (*P* = 0.1).

### Study characteristics and results of individual studies

Data from 1526 patients were collected. 60.8% of those patients (928 of 1526 patients) were female. The mean age of the patients at baseline was 63.9 ± 3.6 years. No difference was found in the sample size, mean age, and percentage of females between non-selective and selective NSAID group at baseline (*P* > 0.5). Generalities of the included studies and the patient demographics are shown in Table 2.

**Table 2:** Generalities and patient demographics of the included studies

Author, year, ref.	Follow-up (months)	Type of treatment	Type of protocol	Samples (*n*)	Mean age (years)	Female gender (%)
Barthel et al. 2002 [17]	12	Meloxicam	7.5 mg daily / 14 days	24	65	42%
		Meloxicam	15 mg daily / 14 days	115	63	65%
		Indomethacin	100 mg daily / 14 days	111	63	64%
Grohs et al. 2007 [7]	12	Rofecoxib	25 mg daily / 7 days	50	60	66%
		Indomethacin	100 mg per daily / 7 days	50	60	60%
Legenstein et al. 2003 [34]	6	Indomethacin	150 mg daily / 12 days	58	68	59%
		Meloxicam	7.5 mg daily / 12 days	58	65	74%
Romano et al. 1992 [35]	24	Indomethacin	100 mg per daily / 20 days	229	62	72%
		Celecoxib	400 mg daily / 20 days	147	59	74%
Saudan et al. 2007 [25]	3	Celecoxib	400 mg daily / 10 days	117	69	53%
		Ibuprofen	1200 mg daily / 10 days	123	70	54%
Van der Heide et al. 2004 [9]	6	Indomethacin	150 mg daily / 7 days	89	67	68%
		Meloxicam	15 mg daily / 7 days	92	67	68%
Van der Heide et al. 2007 [36]	12	Indomethacin	150 mg daily / 7 days	89		62%
		Rofecoxib	50 mg daily / 7 days	85		62%
Winkler et al. 2016 [4]	6	Diclofenac	150 mg daily / 9 days	44	61	45%
		Etoricoxib	90 mg daily / 9 days	45	60	46%

### Results of syntheses

**Fig. 4:**
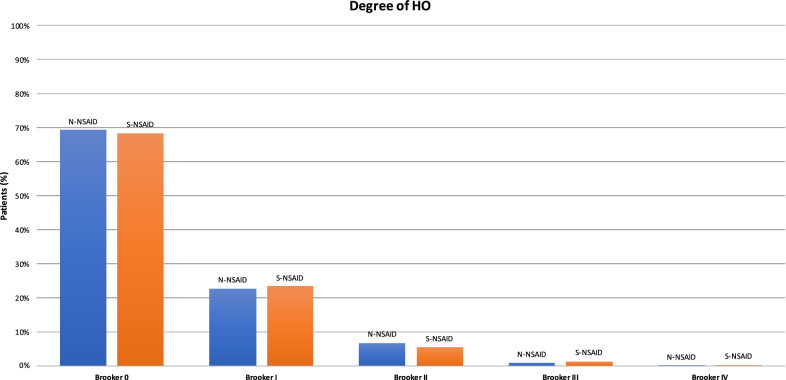
The graph shows that both types of NSAIDs were effective at preventing HO: 72% (1078 of 1502) of patients were classified as Brooker 0, 21% (322 of 1502) Brooker I, 5% (80 of 1502) Brooker II, 1% (16 of 1502) Brooker III, and 0.1% (2 of 1502) Brooker IV

Both selective and non-selective NSAIDs were effective in the prophylaxis of HO (Fig. 4). Both classes of NSAIDs were effective at preventing HO: 72% (1078 of 1502) of patients were classified as Brooker 0, 21% (322 of 1502) as Brooker I, 5% (80 of 1502) as Brooker II, 1% (16 of 1502) as Brooker III, and 0.1% (2 of 1502) as Brooker IV.

No statistically significant difference was found between the selective and non-selective NSAIDs in terms of efficacy (Table 3).

**Table 3:** Comparison of non-selective NSAIDs (N-NSAIDs) versus selective NSAIDs (S-NSAIDs)

Degree of HO	N-NSAID	S-NSAID	OR	95% CI	*I*^2^ (${\varvec{\chi}}$^2^)	*P*
Brooker 0	69.4% (574 of 793)	68.4% (504 of 709)	0.99	0.78 to 1.26	63% (*P* = 0.008)	0.9
Brooker 1	22.7% (164 of 793)	23.4% (158 of 709)	1.04	0.80 to 1.35	45% (*P* = 0.08)	0.8
Brooker 2	6.7% (46 of 793)	5.6% (34 of 709)	0.99	0.59 to 1.67	0% (*P* = 0.8)	0.9
Brooker 3	1.0% (8 of 793)	1.3% (8 of 709)	0.69	0.34 to 0.41	18% (*P* = 0.3)	0.3
Brooker 4	0.1% (1 of 793)	0.3% (1 of 709)	0.61	0.14 to 2.56	0% (*P* = 0.5)	0.5

## Discussion

According to the main findings of the present study, selective and non-selective NSAIDs show similar efficacies when used as prophylaxis for HO following THA. 72% of the patients were classified as Brooker 0, 21% as Brooker I, 5% as Brooker II, 1% as Brooker III, and 0.1% as Brooker IV. However, selective NSAIDs have not been investigated at a large scale, and future high-quality trials are needed to validate these results in a clinical setting. The most commonly investigated drug for the prophylaxis of HO is indomethacin.

Three studies compared indomethacin and meloxicam [9, 17, 34]. Barthel et al. [17] showed that 25% of patients who received meloxicam developed HO, and 10% did so in the indomethacin group. On the contrary, two studies found a similar rate of HO in patients who received indomethacin or meloxicam [9, 34]. Also, celecoxib shows similar efficacy to indomethacin but higher efficacy than ibuprofen in the prevention of HO after THA, with a significantly lower rate of side effects [20, 25]. Very conclusive results came from two studies in which rofecoxib and indomethacin were compared [7, 36], with no significant difference in HO occurrence was observed between the two drugs. Furthermore, Winkler et al. [4] also found similar rates of HO in patients who received etoricoxib and those who received diclofenac. A recent meta-analysis of randomized controlled trials, which included 21 studies and 5995 patients, evaluated the efficacy and safety of NSAIDs for the prevention of HO after THA. The most common N-NSAIDs (indomethacin, ibuprofen, flurbiprofen, ketorolac, diclofenac) and S-NSAIDs (meloxicam, celecoxib, rofecoxib, tenoxicam) were used in the studies included. The authors observed that NSAIDs significantly decreased the occurrence of HO after THA when compared to placebo. However, there were no significant differences in the selective NSAIDs versus non-selective NSAIDs comparison [1].

A prevention protocol of 25 mg indomethacin was administered three times daily for 6 weeks following THA [13]. The same protocol prescribed for only 2 weeks yielded the same efficacy as 6 weeks of therapy [12]. More recently, 50 mg indomethacin administered two or three times daily showed good results for HO prevention [17, 20, 34]. Non-selective NSAID administration for 1 week was effective as well [3, 7, 36], even though some studies revealed a slightly increased risk of HO when the treatment period was shorter than 8 days [3, 16]. Other non-selective NSAIDs used for prophylaxis of HO after THA include ibuprofen and ketorolac. The efficacy of ibuprofen compared to placebo and indomethacin was evidenced in previous reports [37, 38]. The efficacy of ketorolac was analysed in a prospective, double-blind, randomized trial [39]. A total of 152 patients received 60 mg of ketorolac intraoperatively and 30 mg every 8 h for five doses postoperatively; another 151 patients received no prophylaxis for HO. There was significantly less HO in the ketorolac group. None of the patients developed clinically severe HO. A recent Bayesian network meta-analysis that included 26 studies and 6396 THAs demonstrated that prophylaxis with celecoxib was associated with the lowest rate of HO after THA, followed by prophylaxis with diclofenac and naproxen [40]. Celecoxib demonstrated the highest rate of Brooker class 0, followed by diclofenac. Naproxen demonstrated the lowest rate of Brooker I HO, followed by celecoxib. Celecoxib demonstrated the lowest rates of Brooker class II, class III, and class IV HO. On the other hand, tenoxicam, acetylsalicylic acid, and meloxicam were associated with the highest rates of HO following THA.

The present meta-analysis certainly has limitations. The small number of studies included and the heterogeneous drug administration protocols represent the most important limitations of the present study. The high variability in protocols increases the heterogeneity and may bias the conclusion of the present study. We used the modified Brooker staging system to rate the efficacy of the interventions. A limitation of this study is the relatively short length of the mean follow-up, which was shorter than 12 months. We must underline that, although HO formation is generally detectable early after surgery, its extent and Brooker grade cannot be definitively assessed until 12 months after surgery [40]. General health measures included were not reported. We did not consider the surgical approach used in THA in the various studies included, and this may be another risk of bias. The surgical approach used in THA may play an important role [41, 42]. Some studies have been conducted on the influence of the approach used in THA on HO formation [43]. The lowest incidence of HO formation was found after the posterior approach [43, 44]. Zran et al. [45] found a lower incidence of HO in patients undergoing a posterior approach (27.6%) compared to patients undergoing the direct lateral approach (47.7%). Alijanipour et al. [46] compared the direct anterior approach with the direct lateral approach and found a statistically significant greater rate of HO formation with the direct lateral approach. An important strength of our work is the type of study included, as seven of the eight studies were randomized controlled trials. Given these limitations, the results of the present study must be interpreted with caution.

## Conclusion

Selective and non-selective NSAIDs were equally effective for the prevention of HO after THA.

## Data Availability

Not applicable.
